# Age-specific association between thyroid autoimmunity and hypothyroidism in Chinese adults aged over 65 years: a cross-sectional study

**DOI:** 10.3389/fendo.2023.1216308

**Published:** 2023-07-26

**Authors:** Mengjie Zhang, Wenjing Ni, Lina Zhang, Kuanlu Fan, Yu Sun, Chao Liu, Shuhang Xu

**Affiliations:** ^1^ Endocrine and Diabetes Center, The Affiliated Hospital of Integrated Traditional Chinese and Western Medicine, Nanjing University of Chinese Medicine, Nanjing, China; ^2^ Endocrine and Diabetes Center, Jiangsu Province Academy of Traditional Chinese Medicine, Nanjing, China; ^3^ Department of Endocrinology, The Second Affiliated Hospital of Xuzhou Medical University, Xuzhou, China; ^4^ Department of Endocrinology and Metabolism, The Affiliated Suqian Hospital of Xuzhou Medical University, Suqian, China; ^5^ Key Laboratory of Traditional Chinese Medicine Syndrome and Treatment of Yingbing (Thyroid Disease) of State Administration of Traditional Chinese Medicine, Jiangsu Province Academy of Traditional Chinese Medicine, Nanjing, China

**Keywords:** thyroid autoimmunity, subclinical hypothyroidism, overt hypothyroidism, elderly, hypothyroidism

## Abstract

**Background:**

The correlation between thyroid autoimmune (TAI) disease and hypothyroidism in the elderly of different ages remains unclear. This study aimed to investigate the epidemiological characteristics of hypothyroidism, including subclinical hypothyroidism (Shypo) and overt hypothyroidism (Ohypo) in those aged ≥65 years from iodine-adequate areas and reveal the correlation between TAI and hypothyroidism in the elderly of different ages.

**Methods:**

It was a cross-sectional study involving 2,443 subjects aged ≥65 years from two iodine-adequate areas in China by cluster sampling. They were assigned to the 65–69-, 70–79-, and ≥80-year-old age group. All subjects were surveyed by questionnaires and received physical examinations, laboratory testing, and thyroid ultrasound. Epidemiological characteristics of thyroid diseases in the elderly were compared among the three groups. Risk factors for hypothyroidism were predicted by binary logistic regression analysis.

**Results:**

The median urinary iodine level was 238.70 (197.00, 273.70) μg/L. Thyroid peroxidase antibody or thyroglobulin antibody positivity (11.87%) and Shypo (9.13%) were common in the elderly. The prevalence of hypothyroidism in the elderly increases with age. TAI was a risk factor for Shypo (OR, 1.94; 95% CI, 1.35, 2.80; *p* < 0.01) and Ohypo (OR, 7.64; 95% CI, 3.40, 17.19; *p* < 0.01) in elderly Chinese. There was an age-specific correlation between TAI and hypothyroidism in the elderly. However, a significant correlation was not identified between TAI and hypothyroidism in ≥80-year-old age group (*p* > 0.05).

**Conclusion:**

Hypothyroidism, particularly Shypo, is common in the elderly from iodine-adequate areas in China. TAI serves as a risk factor for hypothyroidism in the elderly, with an age-specific correlation with hypothyroidism.

## Introduction

The aging of the global population has accelerated over the past several decades (1). Overall, the incidence of thyroid diseases in the elderly is higher than that in adults (2–6). Our study has also found that hypothyroidism is common in the Chinese elderly, with a prevalence of 10.28% (7).

As the most sensitive index of thyroid function, serum thyroid stimulating hormone (TSH) levels can be influenced by multiple factors like iodine nutrition, age, and autoimmunity (8, 9). In iodine-adequate areas, TSH physiologically increases with age, and the upper limit of TSH for the elderly ≥65 years old is 8.86 mIU/L (10). A recent study from China showed that the TSH level in adults from iodine-excessive areas is significantly higher than that in iodine-abundant areas (2.35 [1.63, 3.44 mIU/L] *vs*. 2.61 [1.78, 4.02 mIU/L]) (11). In addition, our previous study has also confirmed that advanced age and high urine iodine concentration (UIC) are risk factors for hypothyroidism (12).

Thyroid autoimmunity (TAI) is another risk factor for hypothyroidism in iodine-sufficient areas (13). TAI is mainly manifested by elevated levels of thyroid peroxidase antibody (TPOAb) and thyroglobulin antibody (TgAb) (13, 14). TAI has a high incidence that increases with age (15, 16). The National Health and Nutrition Examination Survey (NHANES) has reported that the incidence of TSH >4.5 in the elderly aged 70+ years of the TPOAb-positive group is much higher than that in the TPOAb-negative group (50% *vs*. 10%) (17). However, the association between TAI and thyroid function in the elderly is still controversial, especially in very old people. In the present study, we aimed to analyze epidemiological characteristics of thyroid hypothyroidism, including subclinical hypothyroidism (Shypo) and overt hypothyroidism (Ohypo), in those aged ≥65 years from iodine-adequate areas and reveal the correlation between TAI and hypothyroidism in the elderly of different ages.

## Materials and methods

### Subjects

**Figure 1:**
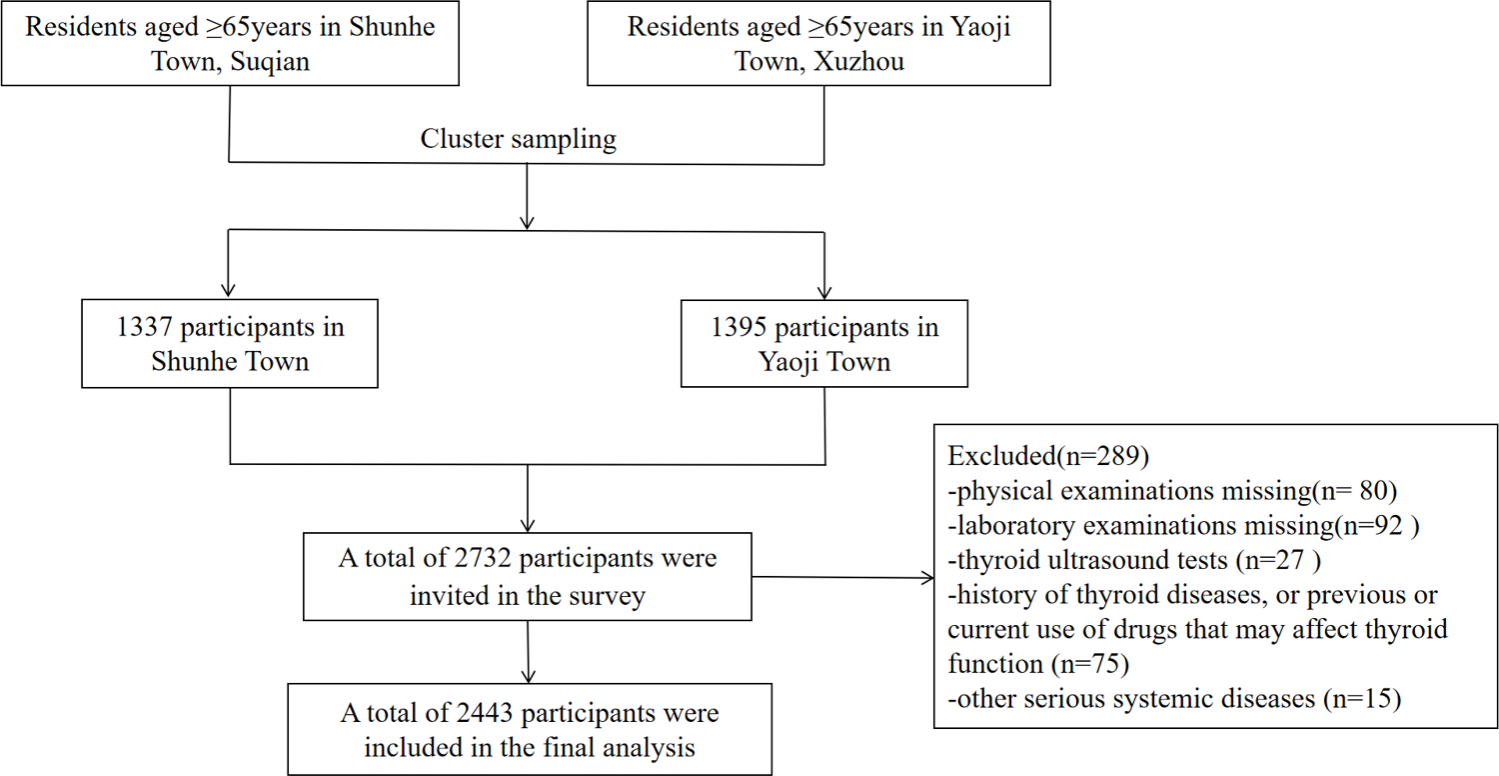
Flow chart of the cross-sectional study.

Thyroid diseases in Older Population: The Screening, Surveillance, and Intervention (TOPS) study was a cross-sectional, population-based study (7). It was approved by the ethics committee of the Jiangsu Provincial Hospital of Integrated Traditional Chinese and Western Medicine (2021-LWKYZ-054). All methods were performed in accordance with the relevant guidelines and regulations. Written informed consent was obtained from all subjects. From May 2021 to August 2021, a total of 2,732 subjects aged ≥ 65 years in Shunhe Town, Suyu District, Suqian City, Jiangsu Province, and Yaoji Town, Suining County, Xuzhou City, Jiangsu Province, were randomly sampled by the cluster sampling method. Excluded were those with (1) a history of thyroid diseases or previous or current use of drugs that may affect thyroid function; (2) other serious systemic diseases; or (3) incomplete clinical data. Finally, 2,443 eligible people aged ≥ 65 years were included (**Figure 1**), involving 1,241 men (50.80%) and 1,202 women (49.20%).

### Methods

All subjects were surveyed by questionnaires and received physical examinations, laboratory testing, and thyroid ultrasound.

#### Questionnaire survey

All subjects were asked to fill out questionnaires, including demographic information (e.g., name, sex, date of birth, telephone, address, etc.), socioeconomic status, medical history (e.g., endocrine system diseases), smoking, drug history, and family history of thyroid diseases and other endocrine system diseases.

#### Physical examinations

The height (Ht) and body weight (Wt) of each subject were recorded to calculate the body mass index (BMI) with the formula: BMI = Wt/Ht^2^ (kg/m^2^). Digital data were recorded with two decimal places.

#### Laboratory testing

After fasting for at least 8 h, venous blood samples (5–10 mL) and urine samples (10–15 mL) collected at 8:00–9:00 a.m. were sent to the Clinical Laboratory, the Affiliated Hospital of Integrated Traditional Chinese and Western Medicine, Nanjing University of Chinese Medicine. Briefly, serum TSH (normal range, 0.27–4.20 μIU/mL), free thyroxine (FT_4_, 12.0–22.0 pmol/L), TgAb (<115.00 IU/mL), TPOAb (<34.00 IU/mL), fasting blood glucose (FBG), and fasting insulin (FINS) were measured by electrochemiluminescence method using a Roche Cobas C702 chemistry analyzer. Serum total cholesterol (TC, <5.20 mmol/L), triglycerides (TG, 0.57–2.26 mmol/L), low-density lipoprotein cholesterol (LDL-c, <3.37 mmol/L), high-density lipoprotein cholesterol (HDL-c, 1.04-3.10 mmol/L), and urinary iodine concentration (UIC, 100–300 μg/L) were measured by colorimetric method using a Roche Cobas C702 chemistry analyzer. Serum 25-hydroxy vitamin D (25[OH]D) was measured by electrochemiluminescence method using a Roche Cobas C602 chemistry analyzer, which was categorized as follows: 25(OH)D deficiency, <12 ng/mL; 25(OH)D insufficiency, 12–20 ng/mL; optimal 25(OH)D level, 20–30 ng/mL; and 25(OH)D sufficiency, ≥30 ng/mL. Glycated hemoglobin (HbA1c, 0%–6.5%) was measured by high-performance liquid chromatography (HPLC) using a Bio-Rad D-10 hemoglobin testing system.

#### Thyroid ultrasound

Thyroid ultrasound was performed by experienced ultrasound physicians using a SIUI Apogee 1000 Neo ultrasound machine at 7.5–13.0 MHz. Briefly, the subject was placed in a supine position to fully expose the neck and asked to stably breathe. Ultrasonic characteristics of the thyroid were examined.

### Diagnostic criteria

The criteria for diagnosis of thyroid dysfunctions were (2, 10) as follows: overt hyperthyroidism (Ohyper), TSH <0.27 μIU/mL, FT4 >22.0 pmol/L, and/or FT3 >6.8 pmol/L; subclinical hyperthyroidism (Shyper), TSH <0.27 μIU/mL and FT3 and FT4 in the reference ranges; Ohypo, TSH >4.20 μIU/mL and FT4 <12.0 pmol/L; and Shypo, TSH >4.20 μIU/mL and FT4 in the reference range. TAI was defined as positive TPOAb or TgAb (13). The criteria for positive thyroid antibodies were as follows: TPOAb >34 IU/mL or TgAb >115 IU/mL. Normal thyroid ultrasonography included: (i) homogeneous glandular echogenicity, (ii) no nodule(s), (iii) no goiter, (iv) no obvious small or absent thyroid gland, and (v) no calcifications or acoustic halo.

Vitamin D status was classified as follows: vitamin D deficiency, <12 ng/mL; vitamin D insufficiency, 12–20 ng/mL; and vitamin D sufficiency, ≥20 ng/mL (18, 19). According to the *Assessment of Iodine Deficiency Disorders and Monitoring Their Elimination: A Guide for Programme Managers (Third Edition)* proposed by World Health Organization, iodine intake was classified as follows: deficient iodine intake, UIC <100 μg/L; adequate iodine intake, 100 μg/L ≤ UIC <200 μg/L; above recommended iodine intake, 200 μg/L ≤ UIC <300 μg/L; and excessive iodine intake, UIC ≥300 μg/L (20).

### Statistical analysis

Statistical analysis was performed by SPSS 22.0. The data were expressed as mean ± standard deviation (SD). Measurement data normally distributed were compared between groups by the two-sample independent *t*-test. Otherwise, they were compared by the Wilcoxon rank-sum test for independent samples. Enumeration data were expressed as constituent ratios. Three or more groups were compared by Chi-square test. Risk factors for Shypo and Ohypo were predicted by binary logistic regression analysis. Two-sided *p* < 0.05 was considered statistically significant.

## Results

### Baseline characteristics of subjects

A total of 2,443 subjects aged ≥65 years were included in the present study, including 1,022 (41.83%) at 65–69 years, 1,095 (44.82%) at 70–79 years, and 326 (13.34%) at ≥80 years. There were 1,202 (49.20%) women and 1,241 (50.80%) men in the study cohort. The median UIC was 238.70 (197.00, 273.70) μg/L, indicating an above-recommended iodine intake.

There were significant differences in the Ht, Wt, BMI, TPOAb, FBG, HbA1c, FINS, TC, TG, LDL-c, and 25(OH)D between men and women (all *p* < 0.01, **Table 1**). No significant differences were found in TSH, TgAb, or HDL-c. In addition, thyroid nodules (30.99%), TPOAb or TgAb positivity (11.87%), and Shypo (9.13%) were common in the elderly. The prevalence of positive TPOAb and positive TgAb was 7.98% and 7.78%, respectively, while Ohypo (1.02%), Ohyper (0.41%), and Shyper (0.25%) were rarely detected. The prevalence of thyroid nodules was significantly higher in women than in men (39.77% *vs*. 22.48%, *p* < 0.01). There were significant differences in the positive rates of TPOAb (6.12% *vs*. 9.90%, *p* < 0.01), TgAb (5.48% *vs*. 10.15%, *p* < 0.01), TPOAb and TgAb (2.89% *vs*. 4.83%, *p* = 0.02), and TPOAb or TgAb (8.62% *vs*. 15.22%, *p* < 0.01) between men and women. We did not detect a significant difference in the prevalence of Shypo, Ohyper, Ohypo, and Shyper (all *p* > 0.05).

**Table 1:** Baseline characteristics of the study population (*n* = 2,243).

	Total (*n* = 2,443)	Male (*n* = 1,241)	Female (*n* = 1,202)	*t*	*p*-value
Age	72.27 ± 5.84	72.23 ± 5.64	72.31 ± 6.04	−0.34	0.73
Ht	1.57 ± 0.09	1.62 ± 0.07	1.51 ± 0.07	39.12	<0.01
Wt	61.16 ± 10.64	64.47 ± 10.35	57.75 ± 9.84	16.45	<0.01
BMI	24.81 ± 3.72	24.49 ± 3.60	25.14 ± 3.82	−4.30	<0.01
TSH	2.75 ± 4.51	2.63 ± 4.94	2.87 ± 4.01	−1.33	0.18
TPOAb	24.64 ± 65.72	20.72 ± 57.25	28.68 ± 73.25	−2.99	<0.01
TgAb	66.06 ± 295.02	59.80 ± 302.03	72.54 ± 287.58	−1.07	0.29
FBG	5.96 ± 1.69	5.86 ± 1.53	6.05 ± 1.83	−2.79	<0.01
HbA1c	6.00 ± 1.00	5.93 ± 0.95	6.07 ± 1.04	−3.41	<0.01
FINS	6.90 ± 6.44	5.86 ± 6.66	7.96 ± 6.03	−8.16	<0.01
TC	4.97 ± 1.06	4.75 ± 0.98	5.19 ± 1.09	−10.38	<0.01
TG	1.42 ± 1.00	1.23 ± 0.92	1.60 ± 1.05	−9.24	<0.01
HDL-c	1.36 ± 0.39	1.35 ± 0.39	1.38 ± 0.39	−1.53	0.13
LDL-c	2.92 ± 0.84	2.80 ± 0.80	3.04 ± 0.87	−7.15	<0.01
25(OH)D	32.21 ± 12.06	37.58 ± 12.02	26.68 ± 9.28	25.14	<0.01
Thyroid diseases					
TPOAb (+)	7.98% (195/2,443)	6.12% (76/1,241)	9.90% (119/1,202)	–	<0.01
TgAb (+)	7.78% (190/2,443)	5.48% (68/1,241)	10.15% (122/1,202)	–	<0.01
TPOAb (+) and TgAb (+)	3.89% (95/2,443)	2.98% (37/1,241)	4.83% (58/1,202)	–	0.02
TPOAb (+) or TgAb (+)	11.87% (290/2,443)	8.62% (107/1,241)	15.22% (183/1,202)	–	<0.01
Ohyper	0.41% (10/2,443)	0.32% (4/1,241)	0.50% (6/1,202)	–	0.71
Shyper	0.25% (6/2,443)	0.16% (2/1,241)	0.33% (4/1,202)	–	0.65
Ohypo	1.02% (25/2,443)	0.64% (8/1,241)	1.41% (17/1,202)	–	0.06
Shypo	9.13% (223/2,443)	9.27% (115/1,241)	8.99% (108/1,202)	–	0.81
Thyroid nodule	30.99% (757/2,443)	22.48% (279/1,241)	39.77% (478/1,202)		<0.01

Ht, height; Wt, weight; BMI, body mass index; TSH, thyroid stimulating hormone; TPOAb, thyroid peroxidase antibody; TgAb, thyroglobulin antibody; FBG, fasting blood glucose; HbA1c, glycated hemoglobin; FINS, fasting insulin; TC, total cholesterol; TG, triglyceride; HDL-c, high-density lipoprotein cholesterol; LDL-c, low-density lipoprotein cholesterol; 25(OH)D, 25-hydroxy vitamin D; Ohyper, overt hyperthyroidism; Shyper, subclinical hyperthyroidism; Ohypo, overt hypothyroidism; Shypo, subclinical hypothyroidism.

### Prevalence of hypothyroidism increases with age

The prevalence of hypothyroidism significantly rose with age in the elderly (65–69 years, 8.02%; 70–79 years, 11.51%; ≥80 years, 12.27%; *p* = 0.01). The trend of increasing prevalence with age was found in Shypo (65–69 years, 7.24%; 70–79 years, 10.14%; ≥80 years, 11.66%; *p* = 0.02) rather than Ohypo (65–69 years, 0.78%; 70–79 years, 1.37%; ≥80 years, 0.61%; *p* = 0.30). No significant differences in the prevalence of thyroid nodules, Shyper, and Ohyper were detected among people aged 65–69 years, 70–79 years, and ≥80 years (all *p* > 0.05, **Table 2**). In addition, the prevalence of positive TPOAb and TgAb slightly rose with age, up to 3.99% in people aged ≥80 years. However, no significant differences in the prevalence of positive TPOAb and TgAb were identified among the three age groups (all *p* > 0.05).

**Table 2:** The prevalence of thyroid dysfunction and thyroid nodule in different age groups (*n* = 2,243).

	65–69 years old	70–79 years old	≥ 80 years old	*p*-value
Total	1,022	1,095	326	
TPOAb (+)	8.32% (85/1,022)	7.49% (82/1,095)	8.59% (28/326)	0.81
TgAb (+)	7.63% (78/1,022)	7.21% (79/1,095)	10.12% (33/326)	0.22
TPOAb (+) and TgAb (+)	3.82% (39/1,022)	3.93% (43/1,095)	3.99% (13/326)	0.97
TPOAb (+) or TgAb (+)	12.13% (124/1,022)	10.78% (118/1,095)	14.72% (48/326)	0.15
Ohyper	0.29% (3/1,022)	0.55% (6/1,095)	0.31% (1/326)	0.63
Shyper	0.29% (3/1,022)	0.09% (1/1,095)	0.61% (2/326)	0.25
Ohypo	0.78% (8/1,022)	1.37% (15/1,095)	0.61% (2/326)	0.30
Shypo	7.24% (74/1,022)	10.14% (111/1,095)	11.66% (38/326)	0.02
Hypo	8.02%(82/1,022)	11.51% (126/1,095)	12.27% (40/326)	0.01
Thyroid nodule	29.94% (306/1,022)	30.59% (335/1,095)	35.58% (116/326)	0.15

TPOAb, thyroid peroxidase antibody; TgAb, thyroglobulin antibody; Ohyper, overt hyperthyroidism; Shyper, subclinical hyperthyroidism; Ohypo, overt hypothyroidism; Shypo, subclinical hypothyroidism; Hypo, hypothyroidism.

### TAI is an independent risk factor for hypothyroidism in the elderly

Binary logistic regression analysis involving gender, age, blood lipid levels, TAI, UIC, and 25(OH)D was performed to identify risk factors for hypothyroidism, including Shypo and Ohypo. It is shown that age (hypothyroidism: OR, 1.04; 95% CI, 1.02–1.06; *p* < 0.01; Shypo: OR, 1.04; 95% CI, 1.02–1.06; *p* < 0.01) and TAI (hypothyroidism: OR, 2.46; 95% CI, 1.76–3.43; *p* < 0.01; Shypo: OR, 1.94; 95% CI, 1.35–2.80; *p* < 0.01) were significantly correlated with hypothyroidism and Shypo in the elderly (**Table 3**). TAI was further identified as an independent risk factor for Ohypo in the elderly (OR, 7.64; 95% CI, 3.40–17.19; *p* < 0.01). The prevalence of hypothyroidism including Ohypo and Shypo in the elderly was not associated with gender, blood lipid levels, iodine intake, and vitamin D status (all *p* > 0.05).

**Table 3:** Risk factors for hypothyroidism (including Ohypo and Shypo) in the elderly analyzed by binary logistic regression (*n* = 2,243).

	Hypothyroidism			Shypo			Ohypo		
	OR	95% CI	*p*-value	OR	95% CI	*p*-value	OR	95% CI	*p*-value
Sex (female)	0.89	[0.67, 1.18]	0.41	0.86	[0.64, 1.16]	0.32	1.26	[0.50, 3.17]	0.62
Age	1.04	[1.02, 1.06]	<0.01	1.04	[1.02, 1.06]	<0.01	1.00	[0.94, 1.07]	0.94
TC	1.22	[0.91, 1.63]	0.18	1.25	[0.93, 1.67]	0.14	0.74	[0.22, 2.51]	0.62
TG	1.05	[0.90, 1.22]	0.54	1.05	[0.90, 1.22]	0.58	1.17	[0.66, 2.10]	0.59
HDL-c	1.02	[0.67, 1.54]	0.94	0.99	[0.64, 1.52]	0.95	1.64	[0.34, 8.00]	0.54
LDL-c	0.95	[0.69, 1.31]	0.76	0.86	[0.62, 1.20]	0.38	2.65	[0.70, 10.01]	0.15
UIC	1.00	[1.00, 1.00]	0.03	1.00	[1.00, 1.00]	0.03	1.00	[1.00, 1.00]	0.48
TAI	2.46	[1.76, 3.43]	<0.01	1.94	[1.35, 2.80]	<0.01	7.64	[3.40, 17.19]	<0.01
25(OH)D <20 ng/mL	0.93	[0.62, 1.40]	0.74	0.86	[0.55, 1.32]	0.48	1.74	[0.64, 4.72]	0.28

TC, total cholesterol; TG, triglyceride; HDL-c, high-density lipoprotein cholesterol; LDL-c, low-density lipoprotein cholesterol; UIC, urinary iodine concentration; TAI, thyroid autoimmunity; 25(OH)D, 25-hydroxy vitamin D; Ohypo, overt hypothyroidism; Shypo, subclinical hypothyroidism.

### The correlation between TAI and hypothyroidism is age-specific in the elderly

**Figure 2:**
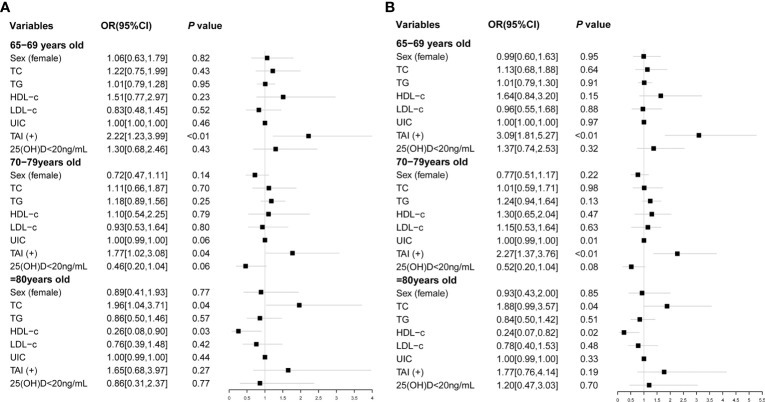
Risk factors of Shypo **(A)** and hypothyroidism **(B)** in different age groups analyzed by binary logistic regression. TC, total cholesterol; TG, triglyceride; HDL-c, high-density lipoprotein cholesterol; LDL-c, low-density lipoprotein cholesterol; UIC, urinary iodine concentration; TAI, thyroid autoimmunity; 25(OH)D, 25-hydroxy vitamin D; Shypo, subclinical hypothyroidism.

We further explored risk factors for hypothyroidism in the elderly in different age groups. It is shown that TAI was a risk factor for hypothyroidism in the same age groups (65–69 years group: OR, 3.09; 95% CI, 1.81–5.27; *p* < 0.01; 70–79 years group: OR, 2.27; 95% CI, 1.37–3.76; *p* < 0.01; **Table 4**; **Figure 2**), as well as Shypo in those at 65–69 years (OR, 2.22; 95% CI, 1.23–3.99; *p* < 0.01) and 70–79 years (OR, 1.77; 95%CI, 1.02–3.08; *p =* 0.04; **Table 5**; **Figure 2**). TAI did not influence the prevalence of hypothyroidism and Shypo in those aged ≥80 years (both *p* > 0.05), but the increase of TC level (hypothyroidism: OR, 1.88; 95% CI, 0.99–3.57; *p =* 0.04; **Table 4**; **Figure 2**; Shypo: OR, 1.96; 95% CI, 1.04–3.71; *p =* 0.04; **Table 5**; **Figure 2**) and HDL-c level (hypothyroidism: OR, 0.24; 95% CI, 0.07–0.82; *p =* 0.02; **Table 4**; **Figure 2**; Shypo: OR, 0.26; 95% CI, 0.08–0.90; *p =* 0.03; **Table 5**; **Figure 2**) are closely related to hypothyroidism and Shypo in the elderly. In addition, the prevalence of hypothyroidism, including Ohypo and Shypo, in the elderly in different age groups was not influenced by gender and vitamin D status (all *p* > 0.05, **Table 4**).

**Table 4:** Risk factors for hypothyroidism in the elderly stratified by age analyzed by binary logistic regression.

	65–69 years old			70–79 years old			≥80 years old		
	OR	95% CI	*p*-value	OR	95% CI	*p*-value	OR	95% CI	*p*-value
Sex (female)	0.99	[0.60, 1.63]	0.95	0.77	[0.51, 1.17]	0.22	0.93	[0.43, 2.00]	0.85
TC	1.13	[0.68, 1.88]	0.64	1.01	[0.59, 1.71]	0.98	1.88	[0.99, 3.57]	0.04
TG	1.01	[0.79, 1.30]	0.91	1.24	[0.94, 1.64]	0.13	0.84	[0.50, 1.42]	0.51
HDL-c	1.64	[0.84, 3.20]	0.15	1.30	[0.65, 2.04]	0.47	0.24	[0.07, 0.82]	0.02
LDL-c	0.96	[0.55, 1.68]	0.88	1.15	[0.53, 1.64]	0.63	0.78	[0.40, 1.53]	0.48
UIC	1.00	[1.00, 1.00]	0.97	1.00	[0.99, 1.00]	0.01	1.00	[0.99, 1.00]	0.33
TAI	3.09	[1.81, 5.27]	<0.01	2.27	[1.37, 3.76]	<0.01	1.77	[0.76, 4.14]	0.19
25(OH)D <20 ng/mL	1.37	[0.74, 2.53]	0.32	0.52	[0.20, 1.04]	0.08	1.20	[0.47, 3.03]	0.70

TC, total cholesterol; TG, triglyceride; HDL-c, high-density lipoprotein cholesterol; LDL-c, low-density lipoprotein cholesterol; UIC, urinary iodine concentration; TAI, thyroid autoimmunity; 25(OH)D, 25-hydroxy vitamin D.

**Table 5:** Risk factors for Shypo in the elderly stratified by age analyzed by binary logistic regression.

	65–69 years old			70–79 years old			≥80 years old		
	OR	95% CI	*p*-value	OR	95% CI	*p*-value	OR	95% CI	*p*-value
Sex (female)	1.06	[0.63, 1.79]	0.82	0.72	[0.47, 1.11]	0.14	0.89	[0.41, 1.93]	0.77
TC	1.22	[0.75, 1.99]	0.43	1.11	[0.66, 1.87]	0.70	1.96	[1.04, 3.71]	0.04
TG	1.01	[0.79, 1.28]	0.95	1.18	[0.89, 1.56]	0.25	0.86	[0.50, 1.46]	0.57
HDL-c	1.51	[0.77, 2.97]	0.23	1.10	[0.54, 2.25]	0.79	0.26	[0.08, 0.90]	0.03
LDL-c	0.83	[0.48, 1.45]	0.52	0.93	[0.53, 1.64]	0.80	0.76	[0.39, 1.48]	0.42
UIC	1.00	[1.00, 1.00]	0.46	1.00	[0.99, 1.00]	0.06	1.00	[0.99, 1.00]	0.44
TAI	2.22	[1.23, 3.99]	<0.01	1.77	[1.02, 3.08]	0.04	1.65	[0.68, 3.97]	0.27
25(OH)D <20 ng/mL	1.30	[0.68, 2.46]	0.43	0.46	[0.20, 1.04]	0.06	0.86	[0.31, 2.37]	0.77

TC, total cholesterol; TG, triglyceride; HDL-c, high-density lipoprotein cholesterol; LDL-c, low-density lipoprotein cholesterol; UIC, urinary iodine concentration; TAI, thyroid autoimmunity; 25(OH)D, 25-hydroxy vitamin D; Shypo, subclinical hypothyroidism.

## Discussion

The high prevalence of thyroid diseases in the elderly has become widely concerning. The present cross-sectional study recruited people aged ≥60 years from two iodine-adequate areas in China, aiming to reveal epidemiological characteristics of thyroid diseases. It was found that TPOAb or TgAb positivity and Shypo were prevalent in the elderly. The overall prevalence of Ohyper in the elderly was relatively low. A previous epidemiological study from 31 provinces of China’s mainland reported that the prevalence of Shypo in the Chinese population aged ≥60 years is up to 35.22%. Moreover, the incidences of positive TPOAb and TgAb are 11.55% and 10.20% in people aged 60–69 years, and 11.41% and 9.85% in those aged ≥70 years, respectively. The prevalence of Ohyper in people aged 60–69 and ≥70 years is 0.65% and 0.47%, respectively (2). Epidemiological data from other countries and regions have revealed that the prevalence of Ohypo and Shypo in people aged >65 years ranges from 1% to 10% and 1% to 15%, respectively (21). A study from the Netherlands has demonstrated that the incidence of positive thyroid antibodies in people aged ≥85 years is about 13% (22). Consistently, our data revealed that thyroid nodules, positive thyroid antibodies, and Shypo were the most common thyroid diseases in people aged >65 years, the prevalence of which was significantly higher in women than in men. The prevalence of Ohyper and Shyper in the elderly, however, was relatively low.

Age, gender, iodine intake, TAI, metabolic disorders, and vitamin D were all remarkably associated with thyroid diseases. Among them, iodine intake, age, and TAI were the most significant factors for thyroid diseases. Long-term excessive iodine intake downregulates the expression of thyrotropin receptors on the surface of thyroid cells and sodium-iodide symporter and also inhibits the activity of type II (D2) deiodinase. Eventually, thyroid function is impaired (23). In this study, interestingly, UIC was not found to be associated with the prevalence of hypothyroidism in the elderly in areas where iodine is more than adequate. Similarly, there was no significant difference in the prevalence of Shypo among the population in iodine-sufficient areas. However, there was a significant positive correlation between iodine excess and Shypo, which was mainly attributed to nonautoimmune Shypo. We have demonstrated that UIC ≥700 μg/L in iodine-sufficient areas in China is also confirmed as a risk factor for Shypo (12). UIC from spot samples has been shown to be a reliable biomarker of recent iodine intake in the population as a whole (24). However, creatinine adjustment of UIC to correct urine volume has been advocated by some authors (25).

Our results showed that age and TAI were significantly correlated with hypothyroidism in the elderly. Aging and TAI increased the risk of Shypo. The prevalence of Shypo in the elderly increased with age, reaching 11.66% in people aged ≥80 years. NHANES has proposed that the 97.5% quantile of TSH increases by 0.03 mU/L with every 1-year increase in age from 12 years (26). The French Endocrine Society consensus guidelines (2019) have consistently reported that the upper limit of the normal reference of TSH increases with age (8). The fixed reference of TSH in clinical testing may increase the detection rate of Shypo in the elderly. Our previous research has established an age-specific range of TSH levels to reduce the possibility of overdiagnosis of Shypo (8). However, according to the age-specific TSH reference range, the detection rate of Shypo was greatly reduced, and the detection rate of Ohypo was too low to be analyzed statistically. As a result, the age-specific TSH reference range was not used to define hypothyroidism in the present study.

Thyroid antibodies are important indicators for TAI and are closely linked with thyroid function. In the present study, the incidence of positive TPOAb or TgAb was 11.87%, significantly higher in women than in men. People with positive thyroid antibodies are more likely to develop thyroid dysfunction (17, 27). Our results found that positive thyroid antibodies increased the risk of hypothyroidism in people aged 65–69 and 70–79 years but did not influence the prevalence of hypothyroidism in people aged ≥80 years. It is indicated that aging may weaken the influence of thyroid antibodies on thyroid function. TAI is considered a physiological condition adaptive to internal and external environmental changes. At a certain age, TAI becomes less influential on thyroid function. A study from the Netherlands involving 64 people aged 85 years has concluded that TPOAb in the elderly has a limited predictive value for thyroid function (22). However, their findings are limited by a small sample size and a short follow-up period. More efforts are required to clarify the influence of TAI on thyroid function in the elderly, especially in very old people.

Thyroid hormones regulate lipid metabolism, and Shypo can cause abnormalities in lipid metabolism. Our study also found that blood lipids are closely related to Shypo in the elderly, especially in TC and HDL-c. A study in China analyzed the relationship between Shypo and blood lipids, finding that for every 1 mIU/L increase in TSH, the TC level increased by 0.0551 mmol/L in middle-aged and elderly subjects (60–69 years old). Similarly, the LDL-c level tended to increase with age for every 1 mIU/L increase in TSH (28). In addition, the study confirmed that the TSH level was significantly higher in patients with metabolic syndrome than in healthy controls. Hypothyroidism has a significant effect on the components of the metabolic syndrome. Patients with Shypo have an increased risk of low HDL-c (28). The high prevalence of dyslipidemia in the elderly is considered a “natural condition.” Both Shypo and dyslipidemia gradually increase with age, suggesting that the relationship between hypothyroidism and dyslipidemia may be affected by aging.

As a vital immunomodulatory factor, the role of vitamin D in thyroid diseases has been extensively highlighted. Mackawy et al. (28) have found an inverse correlation between 25(OH)D and TSH levels, and the prevalence of vitamin D deficiency remains high in patients with hypothyroidism. Low serum vitamin D level is independently associated with high TSH levels and the incidence of thyroid dysfunction (29, 30). A recent population-based study in China has suggested that vitamin D level is significantly lower in patients with Hashimoto thyroiditis (HT) than in non-HT people. Compared with those with sufficient vitamin D levels, significantly lower FT_4_ and higher TSH levels are detected in people with vitamin D insufficiency or deficiency (31). However, we did not identify a significant correlation between vitamin D levels and the prevalence of Shypo or Ohypo, suggesting the limited influence of vitamin D deficiency on thyroid function in the elderly.

Some limitations should be noted. First, it was a cross-sectional study, and we were unable to assess the causal relationship between the above factors and thyroid function in the elderly. Second, we did not include information about the ultrasound aspects correlated with TAI (i.e., inhomogeneous parenchyma, presence of pseudo-nodules, thyroid volume, etc.) as a possible further tool to define the presence or absence of autoimmune thyroid disease. Third, we did not analyze the influence of sunshine exposure, diet, and vitamin D supplement on thyroid diseases in the elderly. Fourth, it is not sufficient to assess iodine status in the elderly by measuring UIC alone. Because UIC is just a reliable biomarker of recent iodine intake for the population, a creatinine adjustment of UIC to correct urine volume is required. Finally, our findings were obtained from epidemiological data in elderly Chinese; therefore, the correlation between vitamin D status and thyroid diseases in young people remains unclear.

Taken together, hypothyroidism, particularly Shypo, is common in the elderly in iodine-adequate areas. TAI is an age-specific risk factor for hypothyroidism in the elderly. A long-term follow-up is needed to further identify the influence of TAI on thyroid function in the elderly and the potential harm of Shypo on the health of old people.

## Data availability statement

The raw data supporting the conclusions of this article will be made available by the authors, without undue reservation.

## Ethics statement

The studies involving human participants were reviewed and approved by Ethics Committee of Jiangsu Provincial Hospital of Integrated Traditional Chinese and Western Medicine. The patients/participants provided their written informed consent to participate in this study. Written informed consent was obtained from the individual(s) for the publication of any potentially identifiable images or data included in this article.

## Author contributions

MZ, WN, and SX developed the research questionnaire and drafted the protocol for this study. LZ, KF, and YS participated in the field investigation for this study. MZ, WN, and LZ were responsible for data collection and analysis. SX, YS, and CL interpreted the results. MZ and WN drafted the manuscript. SX and YS revised it critically for important intellectual content. All authors agreed to take responsibility for the integrity of the data and the accuracy of the data analysis. All authors have approved the final version of the manuscript.

## References

[1] Kontis V Bennett JE Mathers CD Li G Foreman K Ezzati M. Future life expectancy in 35 industrialised countries: projections with a Bayesian model ensemble. Lancet (2017) 389(10076):1323–35. doi: 10.1016/S0140-6736(16)32381-9 PMC5387671 28236464

[2] Li Y Teng D Ba J Chen B Du J He L. Efficacy and safety of long-Term universal salt iodization on thyroid disorders: epidemiological evidence from 31 provinces of mainland China. Thyroid (2020) 30(4):568–79. doi: 10.1089/thy.2019.0067 32075540

[3] Abu-Helalah M Alshraideh HA Al-Sarayreh SA Al Shawabkeh AHK Nesheiwat A Younes N. A cross-sectional study to assess the prevalence of adult thyroid dysfunction disorders in Jordan. Thyroid (2019) 29(8):1052–9. doi: 10.1089/thy.2018.0579 31146635

[4] Petersen M Knudsen N Carle A Andersen S Jorgensen T Perrild H. Increased incidence rate of hypothyroidism after iodine fortification in Denmark: A 20-year prospective population-based study. J Clin Endocrinol Metab (2019) 104(5):1833–40. doi: 10.1210/jc.2018-01993 30551165

[5] Santos Palacios S Llavero Valero M Brugos-Larumbe A Diez JJ Guillen-Grima F Galofre JC. Prevalence of thyroid dysfunction in a Large Southern European Population. Analysis of modulatory factors. The APNA study. Clin Endocrinol (Oxf) (2018) 89(3):367–75. doi: 10.1111/cen.13764 29893010

[6] Roberts L McCahon D Johnson O Haque MS Parle J Hobbs FR. Stability of thyroid function in older adults: the Birmingham Elderly Thyroid Study. Br J Gen Pract (2018) 68(675):e718–26. doi: 10.3399/bjgp18X698861 PMC6145980 30154078

[7] Ni W Zhang M Wang X Li X Wang Q Wang Y. Age-specific serum thyrotropin reference range for the diagnosis of subclinical hypothyroidism and its association with lipid profiles in the elderly population. Sci Rep (2022) 12(1):20872. doi: 10.1038/s41598-022-24182-w 36463291 PMC9719481

[8] Goichot B Raverot V Klein M Vija Racaru L Abeillon-du Payrat J Lairez O. Management of thyroid dysfunctions in the elderly. French Endocrine Society consensus 2019 guidelines. Short version. Ann Endocrinol (Paris) (2020) 81(5):511–5. doi: 10.1016/j.ando.2020.05.002 32446837

[9] Taylor PN Albrecht D Scholz A Gutierrez-Buey G Lazarus JH Dayan CM. Global epidemiology of hyperthyroidism and hypothyroidism. Nat Rev Endocrinol (2018) 14(5):301–16. doi: 10.1038/nrendo.2018.18 29569622

[10] Zhai X Zhang L Chen L Lian X Liu C Shi B. An age-specific serum thyrotropin reference range for the diagnosis of thyroid diseases in older adults: A cross-Sectional survey in China. Thyroid (2018) 28(12):1571–9. doi: 10.1089/thy.2017.0715 30351201

[11] Zhou Z Liu L Jin M Ren B Meng F Wang D. Relationships between the serum TPOAb and TGAb antibody distributions and water iodine concentrations, thyroid hormones and thyroid diseases: a cross-sectional study of 2503 adults in China. Br J Nutr 2022:1–11. doi: 10.1017/S0007114522002367 35876046

[12] Zhang Y Sun Y He Z Xu S Liu C Li Y. Age-specific thyrotropin references decrease over-diagnosis of hypothyroidism in elderly patients in iodine-excessive areas. Clin Endocrinol (Oxf) (2021). doi: 10.1111/cen.14589 34585413

[13] McLeod DS Cooper DS. The incidence and prevalence of thyroid autoimmunity. Endocrine (2012) 42(2):252–65. doi: 10.1007/s12020-012-9703-2 22644837

[14] Wang H Gao H Chi H Zeng L Xiao W Wang Y. Effect of levothyroxine on miscarriage among women with normal thyroid function and thyroid autoimmunity undergoing in vitro fertilization and embryo transfer: A randomized clinical trial. JAMA (2017) 318(22):2190–8. doi: 10.1001/jama.2017.18249 29234808

[15] Hollowell JG Staehling NW Flanders WD Hannon WH Gunter EW Spencer CA. Serum TSH, T(4), and thyroid antibodies in the United States population (1988 to 1994): National Health and Nutrition Examination Survey (NHANES III). J Clin Endocrinol Metab (2002) 87(2):489–99. doi: 10.1210/jcem.87.2.8182 11836274

[16] Hoogendoorn EH Hermus AR de Vegt F Ross HA Verbeek AL Kiemeney LA. Thyroid function and prevalence of anti-thyroperoxidase antibodies in a population with borderline sufficient iodine intake: influences of age and sex. Clin Chem (2006) 52(1):104–11. doi: 10.1373/clinchem.2005.055194 16254196

[17] Surks MI Hollowell JG. Age-specific distribution of serum thyrotropin and antithyroid antibodies in the US population: implications for the prevalence of subclinical hypothyroidism. J Clin Endocrinol Metab (2007) 92(12):4575–82. doi: 10.1210/jc.2007-1499 17911171

[18] Aspray TJ Bowring C Fraser W Gittoes N Javaid MK Macdonald H. National Osteoporosis Society vitamin D guideline summary. Age Ageing (2014) 43(5):592–5. doi: 10.1093/ageing/afu093 25074538

[19] Liao X Zhang Z Zhang H Zhu H Zhou J Huang Q. Application guideline for vitamin D and bone health in adult Chinese (2014 standard edition). Chin J Osteoporos (2014) 20(9):1011–30. doi: 10.3969/j.issn.1006-7108.2014.09.002

[20] World Health Organization. Assessment of iodine deficiency disorders and monitoring their elimination : a guide for programme managers, 3rd ed. World Health Organization. (2007). Available at: https://apps.who.int/iris/handle/10665/43781.

[21] Bensenor IM Olmos RD Lotufo PA. Hypothyroidism in the elderly: diagnosis and management. Clin Interv Aging (2012) 7:97–111. doi: 10.2147/CIA.S23966 22573936 PMC3340110

[22] Du Puy RS Poortvliet RKE Snel M den Elzen WPJ Ballieux B Dekkers OM. Associations of elevated antithyroperoxidase antibodies with thyroid function, survival, functioning, and depressive symptoms in the oldest old: the leiden 85-plus study. Thyroid (2019) 29(9):1201–8. doi: 10.1089/thy.2019.0129 31382845

[23] Calil-Silveira J Serrano-Nascimento C Laconca RC Schmiedecke L Salgueiro RB Kondo AK. Underlying mechanisms of pituitary-thyroid axis function disruption by chronic iodine excess in rats. Thyroid (2016) 26(10):1488–98. doi: 10.1089/thy.2015.0338 27461375

[24] Rohner F Zimmermann M Jooste P Pandav C Caldwell K Raghavan R. Biomarkers of nutrition for development–iodine review. J Nutr (2014) 144(8):1322S–42S. doi: 10.3945/jn.113.181974 PMC4093988 24966410

[25] Pearce EN Caldwell KL. Urinary iodine, thyroid function, and thyroglobulin as biomarkers of iodine status. Am J Clin Nutr (2016) 104 Suppl 3(Suppl 3):898S–901S. doi: 10.3945/ajcn.115.110395 27534636 PMC5004493

[26] Boucai L Hollowell JG Surks MI. An approach for development of age-, gender-, and ethnicity-specific thyrotropin reference limits. Thyroid (2011) 21(1):5–11. doi: 10.1089/thy.2010.0092 21058882 PMC3012447

[27] Ha J Lee J Jo K Lim DJ Kang MI Cha BY. Sex differences in risk factors for subclinical hypothyroidism. Endocr Connect (2018) 7(4):511–22. doi: 10.1530/EC-18-0023 PMC5881431 29514897

[28] Mackawy AM Al-Ayed BM Al-Rashidi BM. Vitamin d deficiency and its association with thyroid disease. Int J Health Sci (Qassim) (2013) 7(3):267–75. doi: 10.12816/0006054 PMC3921055 24533019

[29] Kim M Song E Oh HS Park S Kwon H Jeon MJ. Vitamin D deficiency affects thyroid autoimmunity and dysfunction in iodine-replete area: Korea national health and nutrition examination survey. Endocrine (2017) 58(2):332–9. doi: 10.1007/s12020-017-1425-z 28936757

[30] Kim D. Low vitamin D status is associated with hypothyroid Hashimoto's thyroiditis. Hormones (Athens) (2016) 15(3):385–93. doi: 10.14310/horm.2002.1681 27394703

[31] Chao G Zhu Y Fang L. Correlation between hashimoto's thyroiditis-related thyroid hormone levels and 25-hydroxyvitamin D. Front Endocrinol (Lausanne) (2020) 11:4. doi: 10.3389/fendo.2020.00004 32117049 PMC7034299

